# Tartary Buckwheat Flavonoids and 25-Hydroxyvitamin D_3_ Mitigate Fatty Liver Syndrome in Laying Hens: Association with Cecal Microbiota Remodeling and Lipid Metabolic Homeostasis

**DOI:** 10.3390/ani15152210

**Published:** 2025-07-27

**Authors:** Dongdong Li, Binlong Chen, Yi Zhang, Zengwen Huang, Zhiqiu Huang, Xi Chen, Caiyun Sun, Yunxia Qi, Yaodong Hu, Ting Chen, Silu Wang

**Affiliations:** College of Animal Science, Xichang University, Xichang 615013, China

**Keywords:** tartary buckwheat flavonoids, 25-Hydroxyvitamin D_3_, fatty liver syndrome, laying hen, lipid metabolism

## Abstract

The objective of this experiment was to investigate the effects of tartary buckwheat flavonoids (TBF) and 25-hydroxyvitamin D_3_ (25-OHD) on fatty liver syndrome (FLS) in laying hens. A total of 450 35-wk-old Lohmann laying hens were selected and randomly divided into five groups, with six replicates per treatment and 15 laying hens in each replicate. The control group was fed a corn-soybean meal basal diet. The FLS group was fed a high- energy–low-protein (HELP) diet, and the other three experimental groups were fed HELP diets supplemented with 60 mg/kg TBF, 69 μg/kg 25-OHD, and 60 mg/kg TBF plus 69 μg/kg 25-OHD, respectively. The experiment lasted 8 weeks. The results demonstrated that feeding laying hens with a HELP diet led to a significant accumulation of fat in their livers, liver enlargement and yellowing, as well as a decline in liver antioxidant capacity and an aggravation of inflammation. TBF alone, 25-OHD alone, and their combination had no effect on the laying performance of laying hens fed with a HELP diet. However, 25-OHD significantly enhanced the albumin content, eggshell strength, and eggshell thickness of eggs (*p* < 0.05). Compared with the HELP group, TBF, 25-OHD, or their combination reduced serum LDL-C and TG (*p* < 0.05). The combined treatment further lowered serum NEFA and MDA, enhanced liver SOD activity (*p* < 0.05), and unlike TBF alone (which reduced hepatic TG) or 25-OHD alone (which decreased liver index), reduced both liver index and hepatic TG (*p* < 0.05). Liver gene expression analysis showed that combined TBF and 25-OHD significantly inhibited the expression of fat synthesis-related genes (ACC, FAS, GPAT1, ChREBP1, LXRα, SREBP-1C, SREBP-2, FABP) as well as inflammation-related genes (IL-6, TNF-α, NF-κB, TLR4) (*p* < 0.05). At the phylum level of the cecal microbiota, TBF increased the abundance of *Bacteroidota* (*p* < 0.05), and combined TBF and 25-OHD tended to increase the abundance of *Firmicutes_D*. At the genus level, TBF increased the abundance of *Phocaeicola_A* (*p* < 0.05). Furthermore, TBF, 25-OHD, or their combination reduced the abundance of *Faecalibacterium* (*p* < 0.05). These findings suggest that combined TBF and 25-OHD mitigates FLS in laying hens potentially through remodeling gut microbiota and maintaining lipid metabolic homeostasis.

## Simple Summary

Fatty Liver Syndrome (FLS) in laying hens, characterized by hepatic fat accumulation and metabolic dysfunction, poses a significant challenge to hens’ health and productivity. This study demonstrated that TBF and 25-OHD effectively mitigated FLS by reducing liver triglycerides, enhancing antioxidant capacity, and suppressing fat synthesis and inflammatory gene expression. Furthermore, TBF and 25-OHD modulated the gut microbiota composition. Specifically, they increased the relative abundances of *Firmicutes_D* and *Lactobacillus* while decreasing the abundance of *Faecalibacterium*. These findings highlight the potential of TBF and 25-OHD as functional feed additives, which can improve hepatic health in laying hens affected by FLS.

## 1. Introduction

In the context of globalization, the laying hen industry, as an important part of the livestock industry, has always borne the heavy responsibility of meeting human demand for high-quality protein. Fatty liver syndrome (FLS) in laying hens is a nutritional and metabolic disease that frequently occurs in high-laying hens. The apparent characteristics of FLS in laying hens are a significant decrease in egg production rate, a significant increase in flock mortality, and the patho-anatomical features of hens with abundant abdominal fat reserves, enlarged livers, and a yellowish-brown or yellow color [1]. The prevalence of FLS in laying hens is as high as 16% worldwide, and the disease affects feed intake, egg production, egg weight, and mortality of laying hens, causing great economic losses to the egg industry [2]. Therefore, how to prevent and control the occurrence of FLS in laying hens has become a hot spot of current research.

Plant flavonoids are a class of secondary metabolites present in plants, belonging to polyphenolic compounds, which are widely found in the roots, stems, leaves and fruits of plants such as legumes, herbs, and tea. Studies have shown that flavonoids, as a kind of natural plant functional component, possess various biological characteristics such as antibacterial, anti-inflammatory, antioxidant, enhancing immunity, regulating lipid metabolism, and improving the health of the organism [3,4]. However, the application research of plant flavonoids in poultry production is relatively scarce. Currently, some studies have been conducted on citrus flavonoids [5], mulberry-leaf flavonoids [6], and total flavonoids from Epimedium [7] in poultry. Tartary buckwheat flavonoids (TBF) are natural products extracted from Tartary buckwheat, which mainly contains natural active substances such as rutin and quercetin [8]. The application research of TBF in poultry is relatively scarce. Studies on mice have confirmed that TBF has antioxidant, hypoglycemic, and anti-inflammatory effects [9]. Studies have confirmed that TBF can effectively alleviate the vascular dysfunction and liver damage in mice induced by a high-fructose diet [10]. In addition, TBF can alleviate insulin resistance and liver oxidative stress in mice caused by high levels of oxidized trimethylamine N-oxide [11]. Based on this, we hypothesized that TBF could alleviate FLS in laying hens.

Vitamin D_3_ (VD_3_) is the traditional source of Vitamin D (VD) in laying hens’ diets, and 25-hydroxyvitamin D_3_ (25-OHD) is an intermediate product of VD_3_ metabolism; since 2006, 25-OHD has been widely used in the poultry industry as an alternative source of VD [12]. 25-OHD circulates more easily in the blood and is the main form of circulation in the blood. Compared with VD_3_, 25-OHD adds a hydroxyl group, and its water solubility is enhanced, so its absorption is neither affected by other fat-soluble vitamins, nor dependent on bile secretion and the microcluster structure formed by fat absorption. In addition, 25-OHD bypasses hepatic conversion, shortens the metabolic process of VD_3_ in the organism, and has higher biological activity [13]. The research has found that 25-OHD has a higher biological efficacy in calcium and phosphorus absorption, bone development, and immunity [14,15]. Furthermore, 25-OHD may directly regulate lipid metabolism via vitamin D receptor (VDR) in liver, suppressing lipogenic genes (e.g., SREBP-1c) and promoting fatty acid oxidation [16]. Therefore, we hypothesize that the application of 25-OHD in FLS laying hens is more effective than VD_3_, but no relevant research reports have been found so far.

The objective of this study is to establish a fatty liver syndrome (FLS) model in laying hens using a high-energy–low-protein (HELP) diet, and then to determine whether and how TBF and 25-OHD alone or in combination alleviated FLS in laying hens by measuring production performance, serum parameters, and liver gene expression related to lipid metabolism and gut microbial communities. This research can provide an important theoretical basis and data support for the prevention and mitigation of FLHS in laying hens.

## 2. Materials and Methods

### 2.1. Experimental Design and Animal Management

All experiments were performed in accordance with the Guide for the Care and Use of Laboratory Animals prepared by the Institutional Animal Care and Use Committee of Xichang University (Protocol xcc2022012), China.

In this experiment, we selected 450 35 wk-old Lohmann laying hens of similar weight, and then randomly divided them into five groups. Each treatment group consisted of 6 replicates, with 15 hens per replicate. Each replicate was housed in 5 cages (3 hens per cage), and each cage measured 60 cm in length, 35 cm in width, and 45 cm in height. Treatment hens was uniformly distributed in the layer house to minimize environment effects. Each experimental group was fed with a different treatment diet. The five treatment diets used in the experiment (detailed in Table 1) were as follows: the control diet (basal diet), the high-energy–low-protein (HELP) diet, the HELP diet supplemented with 60 mg/kg Tartary buckwheat flavonoids (TBF), the HELP diet supplemented with 69 μg/kg 25-hydroxyvitamin D_3_(25-OHD), and the HELP diet co-supplemented with 60 mg/kg TBF and 69 μg/kg 25-OHD. The hens were raised in fully enclosed chicken coops, with the indoor temperature maintained at around 24 °C. The lighting duration is 16 h, and continuous ventilation is maintained inside the chicken coop. All hens had free access to water via nipple drinkers and feed via feed troughs. The hens were fed diets in mash form during the experiment (36 to 45 wk of age). The formulas and nutritional levels of the diets are shown in Table 2. The nutrition level of the basic diet in this experiment was prepared according to the feeding management manual of Lohmann laying hens with l phase, The energy level of HELP diet is 3050 kcal/kg, and the protein level is 12%. The TBF used in this experiment was obtained from Fufeng Snoot Biotechnology Co., Ltd. in Baoji City, China, with a purity level 50%. 25-OHD was provided by Shandong Haineng Bioengineering Co., Ltd. in Rizhao City, China, with a purity level 0.05%.

### 2.2. Data Record and Sample Collection

During the experiment, daily laying performance was documented for each replicate, and the death of the hens were recorded daily. Then hen-day egg production (HDEP), average egg weight (AEW), egg mass, feed conversion ratio (FCR), average daily feed intake (ADFI) at the laying stage were calculated. At the 8th wk of the experiment, one laying hen from each replicate was randomly selected, and 30 laying hens in total were fasted for 12 h. Blood was collected from the wing vein and centrifuged at 3000 rpm for 10 min, The upper serum samples were stored at a temperature of −20 °C for future testing purposes. The laying hens were weighed and then slaughtered. Fresh liver and abdominal fat were weighed to calculate the liver index and abdominal fat percentage. The complete liver tissue from all experimental hens underwent systematic photographic documentation. Liver samples were stored at −80 °C prior to RNA extraction, while adjacent tissue sections were fixed in 4% paraformaldehyde for subsequent histopathological assessment.

### 2.3. Egg Quality Analysis

In the 8th week of the experiment, for each repetition, 3 eggs were selected and their quality was measured. An egg multitester (EMT-7300, Robot-mation Co., Ltd., Tokyo, Japan) was used to evaluate Haugh unit, albumen height and yolk color. Eggshell strength was measured using an Eggshell Force Gauge Model II (Robot-mation Co., Ltd.). Eggshell thickness was measured at the large end, equatorial region, and small end of the egg with an Eggshell Thickness Gauge (Robot-mation Co., Ltd.).

### 2.4. Serum Parameters

Serum parameters were determined including serum biochemical parameters and serum antioxidant capacity indexes. Serum total cholesterol (TC), triglycerides (TG), aspartate aminotransferase (AST), alkaline phosphatase (ALP), glucose (GLU), low-density lipoprotein cholesterol (LDL-C), high-density lipoproteincholesterol (HDL-C) were measured by an automatic biochemistry analyzer (Shenzhen Mindray Biomedical Electronics Co., Ltd., BS-460, Shenzhen, China). Serum non-esterified fatty acid (NEFA), malondialdehyde (MDA), superoxide dismutase (SOD), glutathione peroxidase (GSH-Px), catalase, (CAT), and total antioxidant capacity (T-AOC) were detected by a specific assay kit (Nanjing Jiancheng Bioengineering Institute, Nanjing, China).

### 2.5. Liver Antioxidant Capacity Parameters, TG and TC Contents

The levels of MDA, SOD, GSH-Px, CAT, T-AOC, TG, and TC in the liver were determined using the colorimetric method. Specific assay kits from Nanjing Jiancheng Bioengineering Institute (Nanjing, China) were employed for these measurements.

### 2.6. Hematoxylin-Eosin Staining

Hematoxylin-Eosin (H&E) staining was performed following the method described in previous studies [17]. Liver tissues were fixed in 4% paraformaldehyde. After fixation, the tissues were dehydrated, embedded, and sectioned into slices approximately 5 μm thick. Subsequently, the sections were stained with hematoxylin and eosin. A microscope slide scanning imaging system (SQS-600P, Shenzhen Shengqiang Technology Co., Ltd., Shenzhen, China) was used to evaluate pathological changes in the liver.

### 2.7. Real-Time Quantitative PCR

Total RNA was extracted from liver tissues using the Tissue Total RNA Isolation Kit (YEASEN, Shanghai, China), followed by cDNA synthesis using the PrimeScrip™ RT Reagent Kit (Takara Bio, Dalian, China). The primers were designed by using NCBI in Table 3. Real-time PCR was performed using a real-time fluorescence quantitative PCR system (Chengdu LILAI, Chengdu, China) and carried out on the QuantStudio™ 3 instrument (Thermo Fisher Scientific, Waltham, MA, USA). Relative mRNA expression levels were calculated using the 2−ΔΔCt method, with β-actin serving as the housekeeping gene to normalize gene transcription.

### 2.8. 16S rRNA Sequencing

The microbial genomic DNA was extracted from the contents of the hen’s colon for sequencing analysis of the 16S rRNA gene. We used specific primers to amplify the V3-V4 region of the bacterial 16S gene. Subsequently, a DNA library was constructed using standard Illumina reagents and sequenced in dual-end mode on the NovaSeq 6000 platform. The raw sequences were truncated during processing, during which the PCR primers were removed. Sequences with expected errors were removed. Through filtering, denoising, and by joining the read segments, the sequences were grouped into operational taxonomic units (OTUs), and species annotation and abundance analysis were performed to determine the taxonomic composition of the samples. After completing these steps, other analyses were also conducted, such as α-diversity assessment and β-diversity assessment, to reveal the differences between individual samples and among different sample groups.

### 2.9. Statistical Analysis

Statistical analyses of the data from this trial were conducted using SPSS software (version 25.0; IBM Inc., New York, NY, USA). Meanwhile, GraphPad Prism version 8.0.2 (GraphPad Software, La Jolla, CA, USA) was employed for generating figures and formatted to a resolution of over 600 dpi. The data were reported as means along with pooled standard errors of the means (SEM). One way analysis of variance (ANOVA) was performed for significance testing, and a *p* value less than 0.05 was defined as statistically significant.

## 3. Results

### 3.1. Effects of TBF and 25-OHD on Laying Performance of Laying Hens

As shown in Table 4. Compared with the control group, the HELP group exhibited significant reductions in HDEP, AEW, egg mass, and ADFI, and there was a significant increase in the Feed/Egg ratio (*p* < 0.05). Compared with the HELP group, the addition of TBF and 25-OHD alone or in combination had no significant effects on HDEP, AEW, egg mass, and ADFI of the hens. However, compared with the HELP + TBF group, the HELP + TBF + 25-OHD group showed a significant decrease in FCR at 1–4 weeks (*p* < 0.05).

### 3.2. Effects of TBF and 25-OHD on Egg Quality of Laying Hens

The egg quality data are presented in Table 5. Compared with the control group and the HELP group, the addition of 25-OHD to the HELP diet significantly increased albumen height and Haugh unit (*p* < 0.05). There was no significant difference in albumen height and Haugh unit among HELP + TBF, HELP + 25-OHD, and HELP + TBF + 25-OHD groups. The HELP + 25-OHD group had a higher eggshell strength compared to the HELP and HELP + TBF groups (*p* < 0.05), but there was no significant difference compared with the control group and the HELP + TBF + 25-OHD group. The egg weight and shell weight in the control group were significantly higher than those in the other groups (*p* < 0.05), and there were no significant differences among the other groups. There were no significant differences in the yolk color, the yolk weight and the eggshell thickness among all groups.

### 3.3. Effects of TBF and 25-OHD on Serum Biochemical Indices

According to Table 6, The serum levels of LDL-C, TG, and NEFA in the HELP group were significantly higher than those in the control group (*p* < 0.05). When TBF and 25-OHD were added alone or in combination to the HELP diet, the serum levels of LDL-C and TG were significantly reduced (*p* < 0.05), and there was no significant difference compared with the control group. Adding TBF and 25-OHD together to the HELP diet significantly reduced the serum NEFA content (*p* < 0.05). There were no significant differences in serum ALP, AST, GLU, HDL-C, and TC among all groups.

### 3.4. Effect of TBF and 25-OHD on Serum and Liver Antioxidant Indices

**Figure 1:**
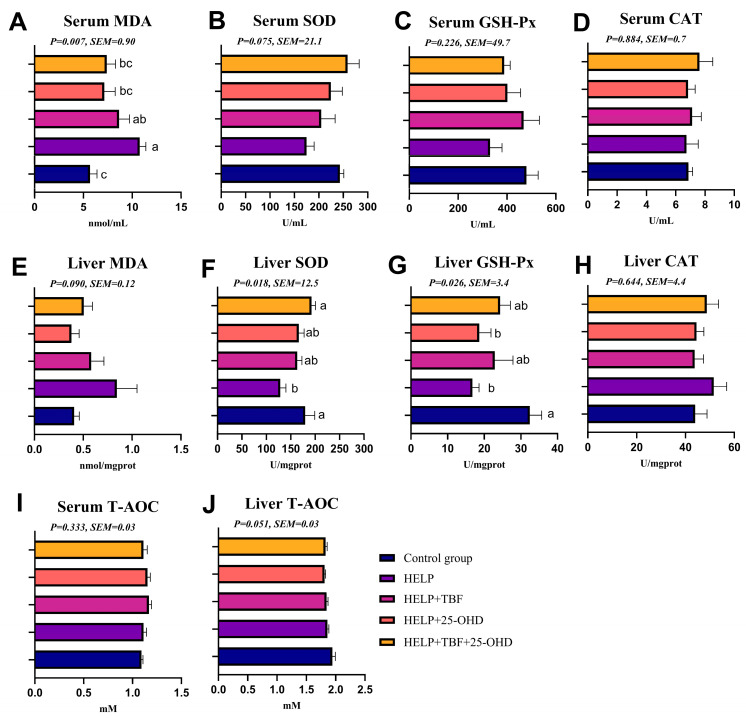
Effect of TBF and 25-OHD on serum and liver antioxidant indices. (**A**–**J**) MDA, SOD, GSH-Px, CAT, T-AOC in serum and liver. Abbreviations: HELP = high-energy–low-protein diet; TBF = Tartary buckwheat flavonoids; 25-OHD = 25-hydroxyvitamin D_3_; MDA = Malondialdehyde; SOD = superoxide dismutase; GSH-Px = glutathione peroxidase; CAT = catalase; T-AOC = total antioxidant capacity. ^a, b, c^. Values without the same letters within the same line indicate a significant difference (*p* < 0.05).

As shown in Figure 1, the serum MDA level in the HELP group was significantly higher than that in the control group (*p* < 0.05). However, supplementing the HELP diet with either 25-OHD or a combination of TBF and 25-OHD significantly reduced the serum MDA level (*p* < 0.05), bringing it to a level comparable to the control group (Figure 1A). Among all groups, no significant differences were observed in the activities of serum SOD, GSH-Px, CAT, and T-AOC (Figure 1B–D,I). Compared with the control group, the activities of SOD and GSH-Px in the liver of the HELP group significantly decreased (*p* < 0.05) (Figure 1F,G). The addition of TBF and 25-OHD together in the HELP diet significantly increased the activity of SOD in the liver (*p* < 0.05) (Figure 1F). There were no significant differences in the levels of liver MDA, CAT, and T-AOC among all groups (Figure 1E,H,I).

### 3.5. Effect of TBF and 25-OHD on Liver Fat Deposition

**Figure 2:**
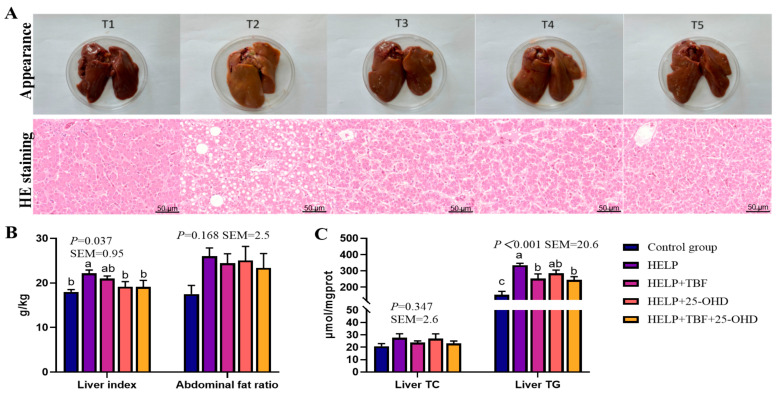
Effect of TBF and 25-OHD on liver fat deposition. (**A**) Liver tissues with H&E staining (Scale bar = 50 μm) and phenotype pictures (T1 = control group; T2 = HELP; T3 = HELP + TBF; T4 = HELP + 25-OHD; T5 = HELP + TBF + 25-OHD). (**B**) Liver index and abdominal fat ratio. (**C**) Liver TC and liver TG. Abbreviations: HELP = high-energy–low-protein diet; TBF = Tartary buckwheat flavonoids; 25-OHD = 25-hydroxyvitamin D_3_; TC = total cholesterol; TG = triglyceride. ^a, b, c^. Values without the same letters within the same line indicate a significant difference (*p* < 0.05).

As presented in Figure 2A, autopsy findings indicated that livers of the control group displayed a reddish-brown hue and had clearly demarcated sharp edges. In contrast, livers from the HELP group exhibited a yellowish tinge, were significantly enlarged, and had a greasy texture. Adding TBF and 25-OHD alone or in combination with the HELP diet alleviated the liver color and swelling. Based on the results of HE staining, the liver cell structure of the laying hens in the control group was intact, while those in the HELP group showed obvious vacuolar degeneration. After adding TBF and 25-OHD alone or in combination with the HELP diet, the vacuolar degeneration of liver cells was alleviated. Furthermore, the liver index in the HELP group was significantly higher than that in the control group. Meanwhile, the liver index in the HELP + 25-OHD group and the HELP + TBF + 25-OHD group was significantly lower (*p* < 0.05) (Figure 2B). There was no significant difference in abdominal fat ratio among all groups. Figure 2C showed that compared with the control group, the liver TG content in the HELP group was significantly increased. The addition of TBF to the HELP diet and the combined addition of TBF and 25-OHD significantly reduced the liver TG content (*p* < 0.05). There was no significant difference in liver TC content among all groups.

### 3.6. Effect of TBF and 25-OHD on the mRNA Expression of Factors Associated with Lipid Metabolism and Inflammation in Hepatic Tissues

**Figure 3:**
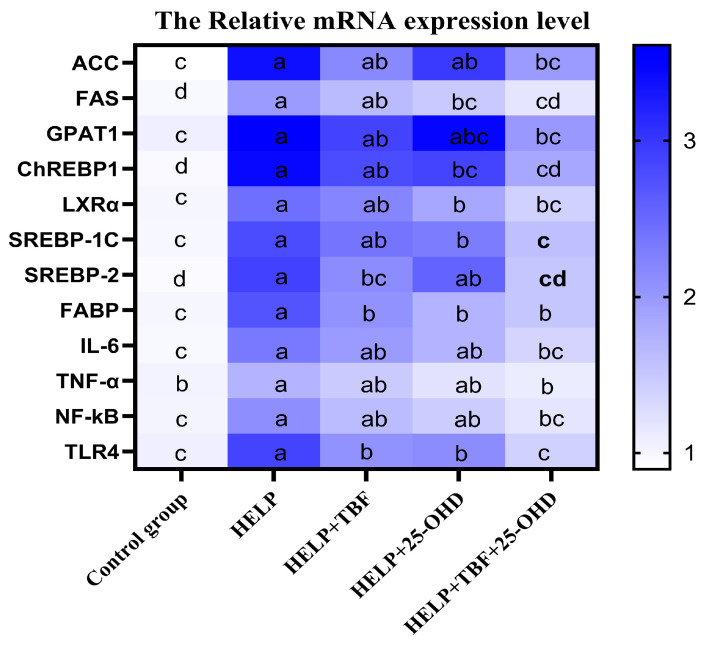
Effect of TBF and 25-OHD on the mRNA expression levels of lipid metabolism-related and inflammatory-related factors in liver. Abbreviations: HELP = high-energy–low-protein diet; TBF = Tartary buckwheat flavonoids; 25-OHD = 25-hydroxyvitamin D_3_; ACC = acetyl CoA carboxylase; ChREBP1 = carbohydrate response element-binding protein 1; FABP = fatty acid-binding protein; FAS = fatty acid synthase; GPAT1 = glycerol-3-phosphate acyltransferase 1; IL-6 = interleukin 6; LXRα = liver X receptor alpha; NF-κB = nuclear factor kappa-B; SREBP-1c = sterol regulatory element-binding protein 1c; SREBP-2 = sterol-responsive element-binding protein 2; TLR4 = toll-like receptors 4; TNF-α = tumor necrosis factor alpha; ^a, b, c, d^. Values without the same letters within the same line indicate a significant difference (*p* < 0.05).

As shown in Figure 3, we measured the related genes expression of de novo lipogenesis, the lipid beta-oxidation, and the inflammatory cytokine from the liver in the laying hens. Compared with the control group, the relative expression levels of Acetyl CoA carboxylase (ACC), fatty acid synthase (FAS), glycerol-3-phosphate acyltransferase 1 (GPAT1), carbohydrate response element-binding protein 1 (ChREBP1), liver X receptor alpha (LXRα), sterol regulatory element-binding protein 1c (SREBP-1c), sterol-responsive element-binding protein 2 (SREBP-2), fatty acid-binding protein (FABP), interleukin 6 (IL-6), tumor necrosis factor alpha (TNF-α), nuclear factor kappa-B (NF-κB), and toll-like receptors 4 (TLR4) in the HELP group were significantly increased; however, when TBF and 25-OHD were added together to the HELP diet, the expression levels of these genes significantly decreased (*p* < 0.05). Compared with the HELP group, the expression levels of SREBP-2, FABP, and TLR4 genes in the HELP + TBF group were significantly decreased, and the expression levels of FAS, ChREBP1, LXRα, SREBP-1c, FABP, and TLR4 genes in the HELP + 25-OHD group were significantly decreased (*p* < 0.05).

### 3.7. Effect of TBF and 25-OHD on the Cecal Microbial Diversity of Laying Hens

**Figure 4:**
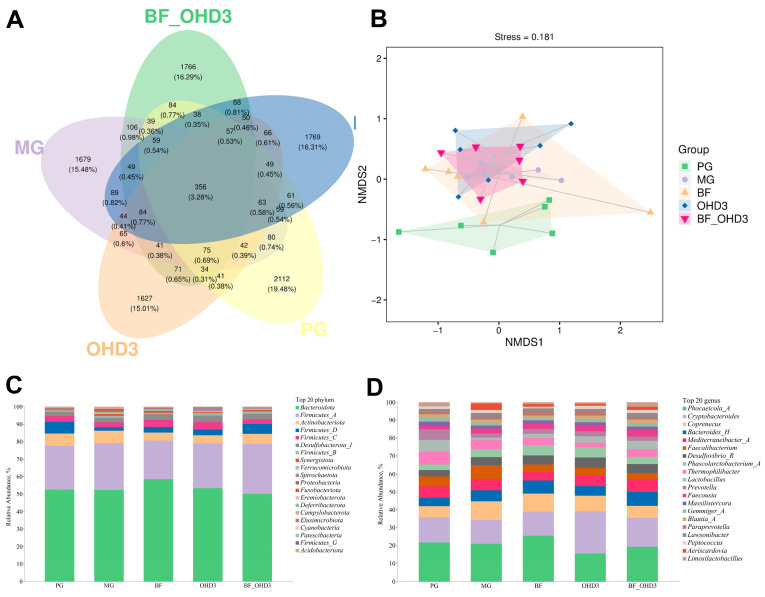
Effect of dietary supplementation of Tartary Buckwheat Flavonoids and 25-hydroxyvitamin D_3_ on the cecal microbiota. (**A**–**D**) display the microbiota composition and diversity analysis results, based on 16S rRNA V3–V4 region sequencing using the NovaSeq 6000 platform. Abbreviations: PG = positive control group; MG = model group (HELP); BF = HELP + Tartary buckwheat flavonoids; OHD3 = HELP + 25-hydroxyvitamin D_3_; BF_OHD3 = HELP + Tartary buckwheat flavonoids + 25-hydroxyvitamin D_3_.

The effects of TBF and 25-OHD on the cecal microbiota of laying hens are summarized in Figure 4A−D and Table 7 and Table 8. It showed that a total of 356 operational taxonomic units (OTUs) were found to share among all five groups, including 2112 unique OTUs in the control group, 1679 unique OTUs in the HELP group, 1769 unique OTUs in the HELP + TBF group, 1627 unique OTUs in the HELP + 25-OHD group, and 1766 unique OTUs in the HELP + TBF + 25-OHD group (Figure 4A). The results of α-diversity analysis indicated that compared with the control group, the Shannon index in the HELP group, the HELP + TBF group, and the HELP + TBF + 25-OHD group significantly increased, and the observed species in the HELP group also significantly increased. However, no significant differences were found in other α-diversity indices (Chao1, Simpson, Pielou_e, and Faith_pd) among the five groups (Table 7). Nonmetric multidimensional scaling (NMDS) analysis indicated significant differences in shape and position between the control group and the other groups (Figure 4B). At the phylum level, the dominant phyla were mainly Bacteroidota, Firmicutes_A, Actinobacteriota, Firmicutes_D, and Firmicutes_C (Figure 4C). The abundance of Bacteroidota was the highest in the HELP + TBF group (*p* < 0.05). Compared with the control group, the abundance of Firmicutes_D decreased in the HELP group, and there was a tendency to increase the abundance of Firmicutes_D when TBF and 25-OHD were added together in the HELP diet (*p* = 0.068) (Table 8). At the bacterial genus level, multiple taxa demonstrated relatively high abundance. These included Phocaeicola_A and Cryptobacteroides, both known to play roles in gut microbiota balance. Additionally, members of Coprenecus, Bacteroides_H, Mediterraneibacter_A, Faecalibacterium, Desulfovibrio_R, Phascolarctobacterium_A, Thermophilibacter, and Lactobacillus were also prominently present (Figure 4D). As shown in Table 8, the statistical analysis results indicated that the abundance of Phocaeicola_A was the lowest in the HELP + 25-OHD group. The addition of TBF and 25-OHD alone or together to the HELP diet significantly reduced the abundance of Faecalibacterium (*p* < 0.05). Moreover, compared with the control group, the abundances of Lactobacillus in the HELP group, HELP + TBF group, and HELP + 25-OHD group significantly decreased (*p* < 0.05). There was no significant difference in the abundance of Lactobacillus between the HELP + TBF + 25-OHD group and the control group.

## 4. Discussion

Widely known as a noninfectious condition, FLS frequently plagues laying hens, adversely affecting their productive performance. Feeding hens with HELP diets has been widely recognized as being able to experimentally induce FLS in hens, and this has been reported in many studies [2,18,19,20,21,22]. Consistent with the findings of this study, previous research has shown that feeding laying hens with the HELP diet leads to a significant decrease in their egg production rate, egg weight, and average egg weight, and also results in a substantial increase in FCR [23]. Furthermore, analysis of our research data indicates that the addition of TBF and 25-OHD to the HELP diet does not result in any enhancement of production performance. A previous study also found that the addition of citrus flavonoids did not enhance egg quality and production performance [5]. In another similar study, adding 0.5 or 2 g of total flavonoids from Rhizoma Drynariae to the diet per kilogram had no effect on the egg production rate, feed intake, and feed conversion ratio of laying hens [24]. The application effect of 25-OHD in laying hens is controversial. Numerous studies have shown that in the diet of laying hens containing VD3, adding 25-OHD additionally does not improve the laying performance and egg quality [12,25,26]. However, the research also found that dietary supplementation with 50 μg/kg of 25-OHD and 2000 IU/kg of VD3 improved eggshell strength, thick albumen height, and the Haugh unit [27]. In this study, supplementing the HELP diet with 25-OHD improved eggshell strength, albumen height, and the Haugh unit. This evidence demonstrates that, specifically for laying hens with FLS, adding 25-OHD to their feed contributes to a notable enhancement in egg quality.

Serum biochemical indices serve as reliable indicators for assessing metabolic status, nutritional state, and physiological or pathological alterations in animals. In this study, it was also found that the contents of LDL-C and TG in the serum of laying hens in the HELP group significantly increased, and the addition of TBF and 25-OHD to the HELP diet could reduce the contents of LDL-C and TG in the serum. Research has demonstrated that plant extracts contain natural molecular compounds that can effectively regulate lipid metabolism in poultry [28]. A recently published study has demonstrated that adding mulberry leaf extract rich in flavonoids to the HELP diet can significantly reduce the levels of TG, low-density lipoprotein and very low-density lipoprotein in laying hens [23]. Polyphenols, which include flavonoids, similarly regulate lipid profiles, as Chang et al. (2025) showed that Myrica rubra pomace polyphenols modulated metabolic markers in diabetic mice, supporting their role in reducing TG and LDL [29]. When 0.2% and 0.4% of green tea catechins were added to the feed of laying hens, it was found that the levels of TC, TG, and LDL in the plasma could be reduced [30]. When adding 100 and 200 g/kg of the flavonoid compound baicalin to broiler diets, it significantly reduced the levels of TC, TG, and LDL-C in their blood serum [31]. At present, there is no direct evidence indicating that 25-OHD has a direct impact on the serum indicators related to lipid metabolism in laying hens. However, considering its roles in calcium and phosphorus metabolism, liver function regulation, and lipoprotein biosynthesis, it may regulate blood lipid levels through indirect pathways. There remains a significant research gap in understanding the regulatory pathways and causal relationship between 25-OHD and serum lipid profiles. Notably, lipid metabolism is governed by complex signaling networks, Chu et al. (2022) revealed that MC-LR exacerbates liver lipid disorders in obese mice via the PI3K/AKT/mTOR/SREBP1 pathway, highlighting potential pathways that 25-OHD might interact with but whose involvement remains uncharacterized [32]. Furthermore, Zhang et al. (2025) identified an association between serum vitamin D levels and cardiovascular disease in type 2 diabetic patients, a condition linked to lipid profile abnormalities, yet the underlying mechanisms connecting vitamin D to lipid regulation remain unclear [33]. Therefore, future investigations should be designed to address this knowledge gap.

In addition, in this study, we demonstrated that supplementing a HELP diet with TBF and 25-OHD not only enhanced the antioxidant capacities of the liver and serum but also reduced the expression of inflammatory factor genes in the liver, with the combined supplementation of the two substances yielding a more pronounced effect. Studies have shown that adding 2 g/kg of Rhizoma Drynariae total flavonoids to the diet can significantly increase the serum T-AOC and GSH-px activities of aged cage-layer hens [24]. The inclusion of dandelion flavonoid extract in the diet significantly elevated the activities of T-AOC, T-SOD, and GSH-Px in the plasma of spent laying hens [34]. The anti-inflammatory effects of flavonoids have been extensively studied. Research has shown that quercetin (a well-known flavonoid) can inhibit the increase in the expression levels of interleukin-1β and TLR-4 in the small intestinal mucosa induced by lipopolysaccharide [35]. Consistent with our findings, earlier studies observed that vitamin D supplementation can attenuate inflammatory markers, particularly under severe inflammatory conditions [36]. The latest research results on sows also indicate that 25-OHD can improve the oxidative stress state in their plasma, enhance the antioxidant capacity of the placenta, and alleviate the oxidative stress condition of the placenta [37].

Nutritional imbalance, especially the imbalance between protein and energy in the feed (similar to what we observed in the HELP feed), is the main cause of FLS [38]. This is supported by findings that low protein (13%) combined with high fat-derived energy (3000 kcal/kg) disrupts lipoprotein assembly (due to insufficient protein for apolipoprotein synthesis), leading to hepatic lipid accumulation and hemorrhage—key features of FLS [39]. As expected, the current research shows that the liver in the HELP group appears pale yellow and is heavily laden with lipid droplets. Birds mainly rely on liver tissue for fat synthesis and metabolism. An increase in fatty acid synthesis and a disruption in fatty acid oxidation and decomposition can lead to fat accumulation in the liver [40]. High-energy diets have been confirmed to induce excessive liver fat deposition (up to 56.66% in dry matter) in laying hens, while natural additives like propolis can reduce liver fat ratio to 28.50–32.00%, mirroring the effects of TBF and 25-OHD observed in this study [41]. The process of fatty acid synthesis from scratch involves three distinct steps: fatty acid synthesis, fatty acid elongation, and subsequently the assembly into triglycerides [42]. Interestingly, in the present study, the dietary co-administration of TBF and 25-OHD was found to effectively alleviate hepatic lipid accumulation and liver injury induced by a HELP diet in laying hens with FLS, this is primarily mediated by downregulating hepatic lipogenic genes including ACC, FAS, GPAT1, ChREBP1, LXRα, SREBP-1c, SREBP-2, and FABP. Previous studies have also shown that adding mulberry leaf flavonoids to the diet can significantly reduce the expression level of SREBP-1c mRNA in the liver of aged laying hens [6]. Similar studies have found that adding 0.8 and 1.2% mulberry leaf extract to the diet of laying hens can significantly reduce the relative expression levels of FASN, PPAR-γ, and SREBP-1c mRNA in the liver [43]. Beyond the lipogenic gene pathways discussed, flavonoids like TBF may also regulate lipid metabolism through bile acid metabolism and intestinal FXR signaling. Cai et al. demonstrated in 2025 that caffeic acid phenethyl ester alleviates obesity via such routes, supporting the potential of related compounds in these pathways [44]. The number of investigations regarding the impacts of 25-OHD on lipid metabolism within the liver of laying hens is relatively limited. Hence, further research in this area is highly warranted.

The gut microbiota plays a pivotal role in maintaining energy homeostasis, regulating lipid metabolism, and safeguarding intestinal health, thereby ensuring the harmonious operation of the gut-liver axis. Imbalances in the gut microbiota are closely associated with liver damage, the development of FLS, and obesity [45]. Recent studies have shown that gut microbial metabolites like *Faecalibacterium prausnitzii*-derived butyric acid can regulate key metabolic enzymes via post-translational modifications—by competitively inhibiting GAPDH lysine 263 lactylation and promoting its butyrylation—thereby reshaping glycolytic metabolism [46]. In the intestinal microbiota of FLS laying hens, it was observed that the abundance of the Proteobacteria phylum increased, while the relative structures of the Firmicutes phylum and the Bacteroidetes phylum changed [47]. Previous studies have also found that in laying hens with severe liver fibrosis and fibrosis syndrome, the number of Firmicutes in their cecum has decreased, the characteristic manifestation is a significant reduction in the abundance of Lactobacillus, while the abundance of *Roseobacter* and *Pseudomonas* has increased [48]. Similarly, in the present study, we observed that, relative to the control group, the abundance of *Firmicutes_D* in the HELP group decreased. Conversely, when TBF and 25-OHD were added to the HELP diet, a tendency towards an increase in the abundance of *Firmicutes_D* was noted. A previous study found that Lactobacillus supplements can improve the development of non-alcoholic fatty liver disease by reorganizing the intestinal microbiota and metabolites [49]. Plant-derived compounds like Litsea cubeba essential oil also regulate gut microbiota, as Chen et al. (2023) reported it increased the abundance of *Oscillospiraceae_UCG-005*, Faecalibacterium, *Blautia*, and *Coprococcus* in pig fecal microflora [50]. Over a period of 35 days, when 45 g/d of mulberry leaf flavonoids were administered to water buffaloes, the abundance of the Proteobacteria phylum in the rumen significantly increased, while the relative abundances of the Actinobacteria phylum, Bacteroidetes phylum and Chlorobacteria phylum decreased [51]. Adding mulberry leaf extract to the HELP diet significantly reduced the relative abundance of Desulfurization bacteria at the phylum level, and significantly increased the relative abundance of Desulfurization bacteria at the genus level [23]. Studies on rats have shown that intraperitoneal injection of 5 μg/kg dose of 1,25(OH)2D3 can reverse the intestinal microbiota imbalance caused by a high-fat diet. This is achieved by increasing the relative abundance of Lactobacillus and reducing the relative abundances of Acetobacter, Rosebacter, and Flavobacter [52]. In this study, we also found that feeding the HELP diet to laying hens significantly reduced the abundance of Lactobacillus; however, adding TBF and 25-OHD concurrently to the HELP diet reversed the decrease in the abundance of Lactobacillus. Such microbiota-regulating effects align with findings by He et al. (2024), who showed that bioactive compounds (graveoline) alleviated liver injury partly by modulating gut microbial balance, including changes in beneficial genera like Lactobacillus [53]. This indicates that the combined use of TBF and 25-OHD can alleviate and prevent FLS in laying hens by regulating the intestinal microbiota of the hens.

## 5. Conclusions

In the present study, we found that supplementation with TBF, 25-OHD, or their combination could effectively mitigate FLS induced by HELP diets in laying hens, with the combined supplementation showing greater efficacy. The observed beneficial effects may be associated with their potential to inhibit hepatic fat formation, regulate gut microbiota composition, and exert anti-inflammatory and antioxidant functions. Future research could explore the molecular regulatory network of their synergism via transcriptomics/proteomics to identify key regulators and core interaction targets, validate causal roles of specific microbiota in FLS improvement through fecal transplantation with metagenomic analysis of functional genes.

## Figures and Tables

**Table 1:** Experimental groups and diet treatments.

Items	Treatment Method
Control group	basal diet
HELP	High-energy–low-protein diet
HELP + TBF	HELP diet with 60 mg/kg TBF
HELP + 25-OHD	HELP diet with 69 μg/kg 25-OHD
HELP + TBF + 25-OHD	HELP diet with 60 mg/kg TBF plus 69 μg/kg 25-OHD

Abbreviations: HELP = high-energy–low-protein diet; TBF = Tartary buckwheat flavonoids; 25-OHD = 25-hydroxyvitamin D_3_.

**Table 2:** Composition and nutrients levels of diets (air-dry basis) %.

Composition of Diet%	Basic Diet	High-Energy–Low-Protein Diet
Corn	61.71	66.88
Soybean meal	26.1	15.75
Soya Oil, GR4	1.71	6.97
DL-Methionine	0.19	0
NaCl	0.3	0.3
Choline Hydrochloride	0.08	0.08
Limestone (coarse)	8.3	8.33
Calcium Hydrophosphate	1.08	1.16
Mineral premix ^1^	0.5	0.5
Vitamin premix ^2^	0.03	0.03
Nutrient level		
ME (kcal/kg)	2700	3050
CP	16.15	12.00
EE	4.07	9.31
Ca	3.5	3.5
Non-Phytate P	0.32	0.32
Lys	0.84	0.57
Met	0.44	0.20

Abbreviations: ME = Metabolizable energy; CP = Crude protein; EE = Ether extract; Ca = Calcium; Lys = Lysine; Met = Methionine. ^1^ Provided per kilogram of diet: Cu (CuSO_4_·5H_2_O) 5 mg, Fe (FeSO_4_·H_2_O) 25 mg, Mn (MnSO_4_·H_2_O) 100 mg, Zn (ZnSO_4_·H_2_O) 60 mg, I (KI) 0.5 mg, Se (Na_2_SeO_3_) 0.2 mg. ^2^ Provided per kilogram of diet: 10,000 IU vitamin A; 1600 IU vitamin D3; 30 mg vitamin E; 3 mg vitamin K_3_; 1 mg vitamin B_1_; 4 mg vitamin B_2_; 3 mg vitamin B_6_; 25 µg vitamin B_12_; 10 mg D-pantothenate; 30 mg niacin acid; 0.5 mg folic acid; 50 µg biotin.

**Table 3:** List of gene primer sequences.

Gene	Forward Primer	Reverse Primer
β-actin	ATCCGGACCCTCCATTGTC	AGCCATGCCAATCTCGTCTT
ACC	ATTATGTCCTGGATAACCTGGTCAAC	TCAGTGTCTTCATTAGTCGCTCAAC
ChREBP1	GATGAGCACCGCAAACCAGAGG	TCGGAGCCGCTTCTTGTAGTAGG
FABP	GAGGCTGACATTACTACTATGGATGG	TTCATTTCCCTTAACTTCTTGCTCATG
FAS	GCATAAATGATACAGAAGTTCCCACAC	CTCTCCACAGGTAATTTCTCGCTTAG
GPAT1	CAGAGGTCAAATAGAAATGGTCAAAGC	GAATGTAAGGAGCAGGTAATCAATGTG
IL-6	GAAATCCCTCCTCGCCAATCTG	CCTCACGGTCTTCTCCATAAACG
LXRα	TGGAGAGACTACAGCACACCTATG	CTTCATTAACATCCGTGGAAACATCAG
NF-kB	TGATGATGATGATGAAGGAATCGTACC	TGCCGCTGCTGCTATGTAGG
SREBP-1C	CAGCAACAGCAGCAGTGACTC	GGTGAGGGCGGTGGGTTC
SREBP-2	CCATTGATTCAGAGCCAGGAAGC	CAAGAGCCACAGGAGGAGAGTC
TLR4	TCCCAACCCAACCACAGTAGC	GAGCAGCACCAATGAGTAGTATAGC
TNF-α	GGACAGCCTATGCCAACAAGTAC	GCGGTCATAGAACAGCACTACG

**Table 4:** Effects of TBF and 25-OHD on laying performance of laying hens.

Items	Control Group	HELP	HELP + TBF	HELP + 25-OHD	HELP + TBF + 25-OHD	SEM	*p*-Value
HDEP, %							
1–4 wk	91.8 ^a^	78.1 ^b^	78.4 ^b^	79.8 ^b^	81.7 ^b^	2.6	0.006
5–8 wk	92.3 ^a^	69.8 ^b^	69.5 ^b^	68.9 ^b^	73.8 ^b^	2.5	0.001
1–8 wk	92.0 ^a^	74.1 ^b^	74.0 ^b^	74.5 ^b^	77.9 ^b^	2.4	<0.001
AEW, g							
1–4 wk	57.2 ^a^	54.8 ^b^	54.4 ^b^	54.3 ^b^	54.6 ^b^	0.44	<0.001
5–8 wk	56.5 ^a^	55.1 ^b^	54.9 ^b^	54.4 ^b^	54.6 ^b^	0.42	0.016
1–8 wk	56.8 ^a^	55.0 ^b^	54.6 ^b^	54.3 ^b^	54.6 ^b^	0.39	<0.001
ADFI, g							
1–4 wk	96.7 ^a^	86.8 ^b^	89.4 ^b^	87.2 ^b^	86.9 ^b^	1.8	0.002
5–8 wk	99.9 ^a^	85.0 ^b^	83.0 ^b^	83.3 ^b^	85.5 ^b^	2.5	<0.001
1–8 wk	98.3 ^a^	85.9 ^b^	86.2 ^b^	85.2 ^b^	86.2 ^b^	1.8	<0.001
FCR							
1–4 wk	1.84 ^c^	2.04 ^ab^	2.11 ^a^	2.02 ^ab^	1.95 ^bc^	0.05	0.011
5–8 wk	1.92 ^b^	2.24 ^a^	2.19 ^ab^	2.23 ^a^	2.13 ^ab^	0.09	0.151
1–8 wk	1.88 ^b^	2.13 ^a^	2.15 ^a^	2.11 ^a^	2.03 ^ab^	0.06	0.035
Egg Mass, g/d							
1–4 wk	52.5 ^a^	42.8 ^b^	42.6 ^b^	43.3 ^b^	44.6 ^b^	1.3	<0.001
5–8 wk	52.2 ^a^	38.5 ^b^	38.0 ^b^	37.5 ^b^	40.3 ^b^	1.4	<0.001
1–8 wk	52.3 ^a^	40.7 ^b^	40.4 ^b^	40.5 ^b^	42.5 ^b^	1.3	<0.001

Abbreviations: HELP = high-energy–low-protein diet; TBF = Tartary buckwheat flavonoids; 25-OHD = 25-hydroxyvitamin D3; HDEP = hen-day egg production; AEW = average egg weight; ADFI = average daily feed intake; FCR: feed conversion ratio (total feed intake/total egg weight). ^a, b, c^. In the same row, different superscript lowercase letters indicate significant differences (*p* < 0.05).

**Table 5:** Effects of TBF and 25-OHD on egg quality.

Items	Control Group	HELP	HELP + TBF	HELP + 25-OHD	HELP + TBF + 25-OHD	SEM	*p*-Value
Egg weight, g	58.5 ^a^	55.1 ^b^	54.8 ^b^	55.6 ^b^	55.5 ^b^	0.9	0.037
Albumen height, mm	6.3 ^b^	6.4 ^b^	7.0 ^ab^	8.0 ^a^	7.1 ^ab^	0.4	0.049
Haugh unit	79.1 ^b^	79.1 ^b^	84.4 ^ab^	90.3 ^a^	85.2 ^ab^	2.7	0.036
Yolk color	5.0	5.2	5.3	5.3	5.5	0.3	0.796
Yolk weight, g/egg	26.8	27.6	27.9	27.6	28.0	0.7	0.757
Eggshell strength, kg/cm^2^	4.95 ^ab^	4.57 ^b^	4.67 ^b^	5.56 ^a^	5.09 ^ab^	0.22	0.037
Eggshell thickness, mm	0.362	0.354	0.336	0.358	0.355	0.008	0.208
Shell weight, g/egg	12.0 ^b^	12.8 ^a^	12.8 ^a^	13.1 ^a^	13.0 ^a^	0.3	0.035

Abbreviations: HELP = high-energy–low-protein diet; TBF = Tartary buckwheat flavonoids; 25-OHD = 25-hydroxyvitamin D_3_. ^a, b^. In the same row, different superscript lowercase letters indicate significant differences (*p* < 0.05).

**Table 6:** Effects of TBF and 25-OHD on serum biochemical indices.

Items	Control Group	HELP	HELP + TBF	HELP + 25-OHD	HELP + TBF + 25-OHD	SEM	*p*-Value
ALP, U/L	543.2	623.0	568.9	499.1	560.9	82.9	0.880
AST, U/L	199.6	247.6	234.7	200.6	203.9	1.14	0.184
GLU, mmol/L	8.62	8.88	9.86	9.98	9.78	0.52	0.249
HDL-C, mmol/L	0.357	0.493	0.57	0.697	0.620	0.08	0.073
LDL-C, mmol/L	0.458 ^b^	1.020 ^a^	0.680 ^b^	0.485 ^b^	0.562 ^b^	0.10	0.005
TC, mmol/L	3.32	3.51	2.96	2.52	2.85	0.39	0.436
TG, mmol/L	9.05 ^b^	14.49 ^a^	7.16 ^b^	7.19 ^b^	7.36 ^b^	1.39	0.004
NEFA, μmol/L	253.7 ^b^	408.6 ^a^	374.2 ^a^	362.9 ^ab^	289.1 ^b^	37.9	0.045

Abbreviations: HELP = high-energy–low-protein diet; TBF = Tartary buckwheat flavonoids; 25-OHD, 25-hydroxyvitamin D3; ALP = alkaline phosphatase; AST = glutamic oxalacetic transaminase; GLU = glucose; HDL-C = high-density lipoproteincholesterol; LDL-C = low-density lipoprotein cholesterol; TC = total cholesterol; TG = Triglyceride; non-esterified fatty acid = NEFA. ^a, b^. In the same row, different superscript lowercase letters indicate significant differences (*p* < 0.05).

**Table 7:** Alpha diversity of different index among all groups.

Items	Control Group	HELP	HELP + TBF	HELP + 25-OHD	HELP + TBF + 25-OHD	SEM	*p*-Value
Chao1	656.718	787.442	712.494	692.556	743.645	30.637	0.058
Simpson	0.981	0.989	0.990	0.989	0.988	0.003	0.433
Shannon	7.628 ^a^	8.163 ^b^	8.073 ^b^	7.936 ^ab^	7.995 ^b^	0.118	0.040
Pielou_e	0.824	0.857	0.859	0.848	0.847	0.011	0.248
Observed_species	616.650 ^a^	745.250 ^b^	675.367 ^ab^	662.667 ^ab^	694.533 ^ab^	27.994	0.047
Faith_pd	57.313	62.636	57.390	58.201	59.865	2.280	0.446
Goods_coverage	0.987	0.985	0.988	0.988	0.985	0.001	0.221

Abbreviations: HELP = high-energy–low-protein diet; TBF = Tartary buckwheat flavonoids; 25-OHD = 25-hydroxyvitamin D_3_. ^a, b^. In the same row, different superscript lowercase letters indicate significant differences (*p* < 0.05).

**Table 8:** Effect of dietary supplementation of TBF and 25-OHD on relative abundances of cecal microflora at phylum and genus levels of laying hens.

Items	Control Group	HELP	HELP + TBF	HELP + 25-OHD	HELP + TBF + 25-OHD	SEM	*p*-Value
Phylum level							
Bacteroidota	0.519 ^a^	0.514 ^a^	0.574 ^b^	0.526 ^ab^	0.482 ^a^	0.017	0.021
Firmicutes_A	0.249	0.264	0.215	0.253	0.274	0.016	0.145
Actinobacteriota	0.070	0.069	0.045	0.045	0.053	0.013	0.458
Firmicutes_D	0.068	0.018	0.032	0.034	0.055	0.005	0.068
Firmicutes_C	0.033	0.029	0.041	0.041	0.026	0.005	0.303
Genus level							
Phocaeicola_A	0.138 ^bc^	0.119 ^ab^	0.157 ^c^	0.097 ^a^	0.112 ^ab^	0.01	0.005
Cryptobacteroides	0.090	0.076	0.083	0.147	0.093	0.026	0.364
Coprenecus	0.039	0.059	0.062	0.054	0.039	0.01	0.403
Bacteroides_H	0.030	0.034	0.045	0.033	0.045	0.008	0.545
Mediterraneibacter_A	0.040	0.036	0.029	0.039	0.042	0.007	0.783
Faecalibacterium	0.036 ^bc^	0.044 ^c^	0.026 ^ab^	0.025 ^ab^	0.019 ^a^	0.004	0.008
Desulfovibrio_R	0.022	0.025	0.031	0.036	0.029	0.009	0.851
Phascolarctobacterium_A	0.020	0.026	0.036	0.036	0.023	0.005	0.099
Thermophilibacter	0.046	0.028	0.023	0.017	0.025	0.007	0.076
Lactobacillus	0.041 ^b^	0.007 ^a^	0.016 ^a^	0.021 ^a^	0.027 ^ab^	0.006	0.011

Abbreviations: HELP = high-energy–low-protein diet; TBF = Tartary buckwheat flavonoids; 25-OHD = 25-hydroxyvitamin D_3_. ^a, b, c^. In the same row, different superscript lowercase letters indicate significant differences (*p* < 0.05).

## Data Availability

Data is available upon request from the corresponding authors.
